# Future of sepsis therapies

**DOI:** 10.1186/s13054-016-1274-9

**Published:** 2016-04-22

**Authors:** Tom van der Poll

**Affiliations:** Academic Medical Center, University of Amsterdam, Division of Infectious Diseases & Center of Experimental and Molecular Medicine, Meibergdreef 9, Room G2-129, 1105 AZ Amsterdam, The Netherlands

**Keywords:** Sepsis, Therapy, Biomarker

Sepsis is defined as life-threatening organ dysfunction caused by a dysregulated host response to infection [1]. In the past decades many clinical trials tested immune modulatory compounds designed to restore homeostasis in patients with sepsis [2, 3]. In spite of these efforts, costing hundreds of millions of dollars, not a single new drug was integrated into clinical practice. Thus, it is obvious that the clinical and scientific communities need to reconsider the therapeutic approach to sepsis. Novel strategies to treat sepsis face serious challenges in their path to the patient in the intensive care unit (ICU).

The first question one might ask when confronted with the many negative sepsis trials is whether our current understanding of the pathophysiology of sepsis is correct. The traditional concept of sepsis as a syndrome caused by uncontrolled injurious inflammation has been replaced by the current model of a multifaceted host response, entailing not only abundant and sustained inflammation, but also lengthy immune suppression [4, 5]. It is now widely acknowledged that acute preclinical sepsis models do not adequately capture the prolonged course in patients with sepsis, in whom the majority develop organ dysfunction over the course of days with deaths occurring mostly more than 1 week after ICU admission. Preclinical research on novel therapeutic interventions should better integrate current knowledge of the course and consequences of sepsis, incorporating aged animals with comorbidity and supportive care, including resuscitation and antibiotic therapy, in different models relevant for sepsis.

A second challenge results from the heterogeneity of sepsis and the patients who are affected. Patients with sepsis are quite heterogeneous, not only with respect to the source of infection and causative pathogen, but also with regard to age, genetic composition, comorbidities, chronic medication, and life style. As a consequence, patients with sepsis have a wide variability in their risk of death and in the absolute benefit that they can derive from a given therapy. Considering the complexity of the host response to sepsis, it is difficult to imagine that a drug targeting one host mediator will provide benefit to all sepsis patients.

What can be done to discover novel therapeutic targets and to improve trial design for testing new interventions? Adequate identification of drug targets should make use of extensive preclinical research including both cellular assays and a combination of animal models relevant for sepsis. Importantly, the possible involvement of pathways implicated in the outcome of experimental sepsis should be verified in patients with sepsis by detailed measurements over time. This research can also be used to develop assays for sets of biomarkers that provide insight into the activity of the targeted pathway; such assays can then be used for inclusion of patients and to monitor the effect of the intervention. This would address two serious problems associated with sepsis trials performed in the last decades. First, a biomarker-guided inclusion of patients is more likely to identify patients that might benefit from a targeted therapy than the traditional inclusion of patients based on clinical criteria. Second, such an approach likely results in more adequate dosing of novel drugs, since this would be guided by measurement of specific biological effects rather than by animal data and relatively limited pharmacokinetic studies in humans. Systems biology is expected to be valuable in identifying sets of biomarkers and their use in what has been called “personalized medicine”. In this respect, a very recent manuscript describing two distinct host response types in patients with severe community-acquired pneumonia, based on analyses of the blood leukocyte transcriptome, is of major interest; one host response type was associated with a clear immune suppressive phenotype and increased mortality [6]. The authors reported a set of seven genes that adequately discriminated the two response types; these genes could be easily incorporated in a polymerase chain-based bedside test to be used for the identification of patients who might benefit (or not) from immune stimulatory therapy. Evidence that the efficacy of a drug can be determined by the type of host response was provided by a re-analysis of the pivotal trial with recombinant human interleukin-1 receptor antagonist in patients with sepsis, showing that—while the intervention overall had no effect—it strongly improved survival in the subgroup of patients with signs of a macrophage activation syndrome [7].

Another point of attention comes from the question how to measure the success of a novel therapeutic. Since the case fatality rate of sepsis has decreased [8, 9], the long-term morbidity of sepsis has received increasing attention [10]. End points beyond the traditional 28-day mortality can capture late physical and cognitive sequelae, and could alter the focus for drug development, moving away from attempts to modify the early course of the host response and instead seeking to support faster and more complete recovery.

Evidence is emerging that non-infectious critical illness can be associated with similar disturbances in host-response pathways as observed in sepsis, especially after a prolonged stay on the ICU [11]. Some critically ill patients without infection might benefit from similar immune modulatory therapies as patients with sepsis, yet they have been systemically excluded from sepsis trials. From a theoretical perspective, it is worthy to evaluate drugs targeting a specific host-response pathway in critically ill patients in whom that pathway is disturbed irrespective of the presence of infection as a triggering event.

It is time to implement new knowledge and technology in the clinical evaluation of new sepsis treatments. The challenge for the coming years will be to translate the rapidly increasing understanding of the molecular pathophysiology of sepsis into new drugs to be tested in only those patients with sepsis (or non-infectious critical illness) in whom the targeted pathway is derailed, making use of rapid tests that can also monitor the drug effect in time. The technology to manufacture bedside molecular tests with very limited hands-on time is there, we now need the intellectual contents with which these tests can be filled. This approach, possibly combined with alternative trial designs and/or end points, may deliver the positive sepsis trials and the clinical implementation of new sepsis strategies for which researchers and clinicians have waited for a long time.

## Abbreviation

ICU: intensive care unit
